# Riddled with holes: Understanding air space formation in plant leaves

**DOI:** 10.1371/journal.pbio.3001475

**Published:** 2021-12-06

**Authors:** Christopher D. Whitewoods

**Affiliations:** Sainsbury Laboratory, University of Cambridge, Cambridge, United Kingdom

## Abstract

Plants use energy from sunlight to transform carbon dioxide from the air into complex organic molecules, ultimately producing much of the food we eat. To make this complex chemistry more efficient, plant leaves are intricately constructed in 3 dimensions: They are flat to maximise light capture and contain extensive internal air spaces to increase gas exchange for photosynthesis. Many years of work has built up an understanding of how leaves form flat blades, but the molecular mechanisms that control air space formation are poorly understood. Here, I review our current understanding of air space formation and outline how recent advances can be harnessed to answer key questions and take the field forward. Increasing our understanding of plant air spaces will not only allow us to understand a fundamental aspect of plant development, but also unlock the potential to engineer the internal structure of crops to make them more efficient at photosynthesis with lower water requirements and more resilient in the face of a changing environment.

## Abstract 2

Leaves are interwoven with large air spaces to increase the efficiency of photosynthesis; however, how these air spaces form and how different patterns have evolved is almost unknown. This Unsolved Mystery article discusses the existing evidence and poses new avenues of research to answer this question.

## Introduction

**Fig 1:**
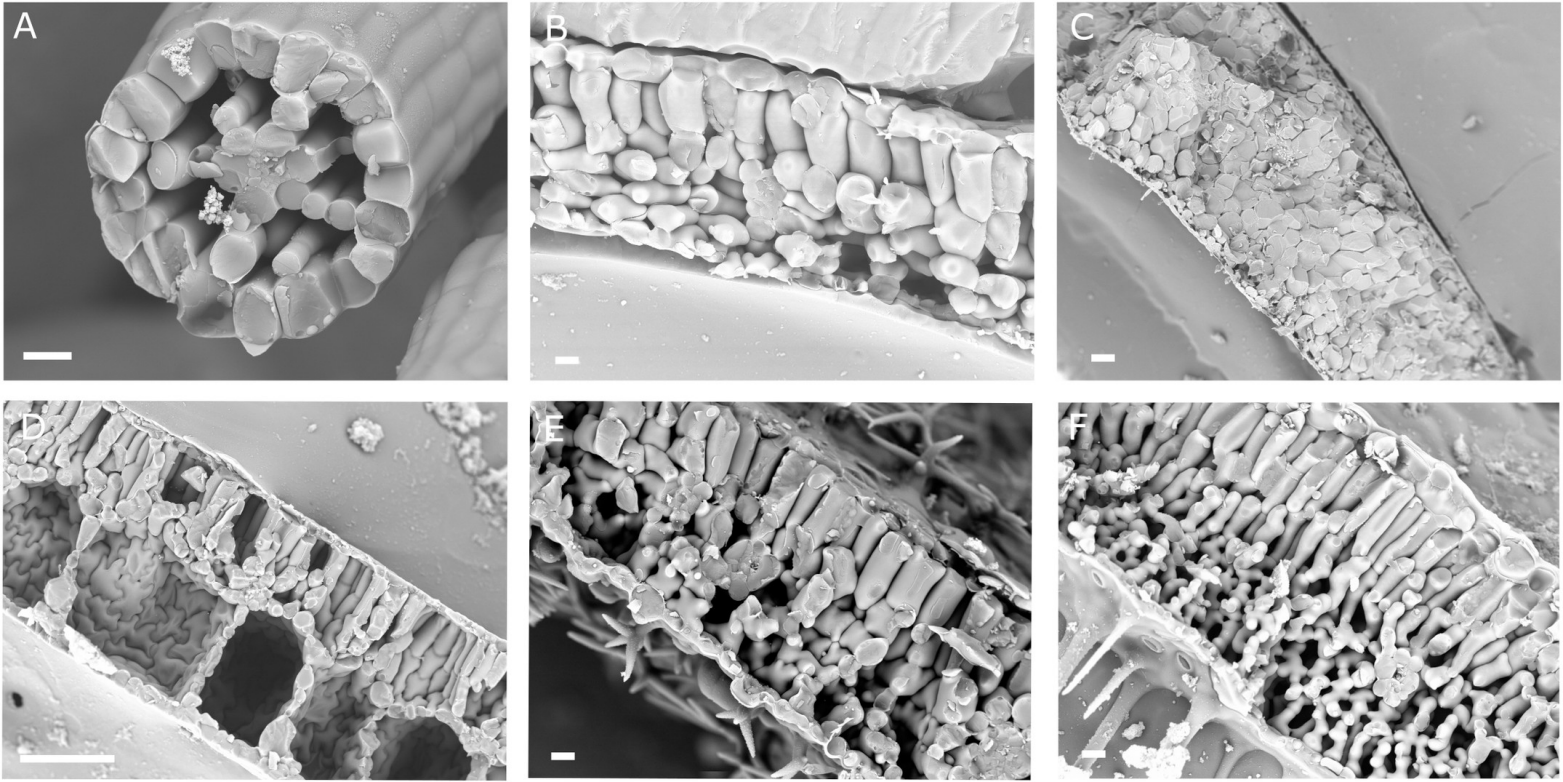
Leaf air space patterns vary between species. Freeze fracture scanning electron micrographs of leaf air spaces in the aquatic *Utricularia gibba* **(A)**, the mesophyte *Arabidopsis thaliana* **(B)**, the succulent xerophyte *Aeonium arboreum* **(C)**, the aquatic *Hydrocharis morsus-ranae* **(D)**, and the mesophytes *Lavandula angustifolia* **(E)** and *Verbena bonariensis* **(F)**. All leaves are arranged with the adaxial (upper) surface upmost, apart from *U*. *gibba* (A), which is radial. Scale bars are 20 μm in A, B, E, and F and 200 μm in C and D.

Plants are made of air. Not only do they build their bodies from carbon molecules in the atmosphere, but many of their tissues are interwoven with air spaces (Fig 1). In leaves, these intercellular air spaces form up to 70% of leaf volume [1], and they are also present in the roots and stems of many species.

These air spaces serve many functions. Leaf air spaces increase the efficiency of gas exchange and were a key innovation that allowed plants to colonise the land [2]. Leaf air space architecture also controls photosynthetic capacity and water use efficiency, and air spaces in roots (aerenchyma) confer resilience to flooding [3]. Modified air spaces are also central plant adaptations to changing environments. Succulent plants in arid environments contain reduced air spaces (e.g., Fig 1C), and enlarged, highly patterned air spaces evolved over 200 times as land plants independently moved back into the water to become aquatic (Fig 1A and 1D) [4]. In this context, air spaces enable efficient gas exchange underwater and allow plants to float and efficiently compete for light.

Decades of work has characterised the arrangement of air spaces in many different plant structures [5–8], and recent advances in microscopy and genetics have highlighted the complexity of this arrangement in 3 dimensions and demonstrated that air space patterning is functionally important [9,10]. However, despite being a fundamental part of plant structure and function, how air spaces develop and evolve is a relative mystery, especially in leaves. In this article, I highlight the problem of how air spaces form in leaves by asking 3 questions: (1) How do leaf air spaces form? (2) What molecular mechanisms control and pattern leaf air space formation? and (3) How have these mechanisms been modified to evolve new leaf air space arrangements?

### How do leaf air spaces form?

**Fig 2:**
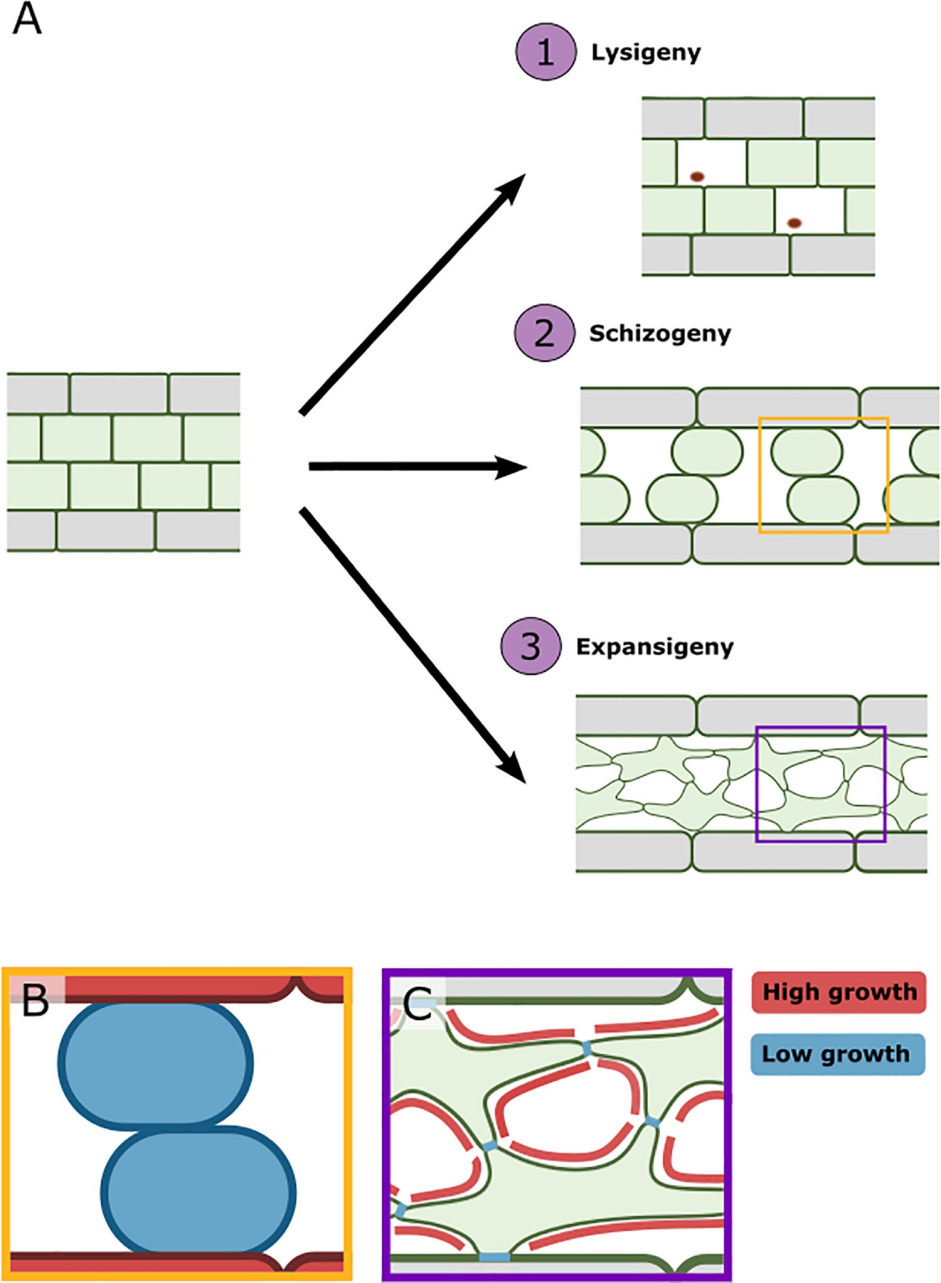
Differential growth underlies air space formation by schizogeny and expansigeny. **(A)** Air spaces can form in 3 ways: (1) lysigeny, where cells die to produce holes within a tissue, often leaving cellular debris behind (red spots, A1); (2) schizogeny, where cells are physically separated (A2); and (3) expansigeny, where cell walls adjacent to air spaces preferentially expand compared to cell walls touching neighbouring cells (A3). In schizogeny, higher growth in the epidermis pulls slower growing internal cells apart (yellow box in A2 and B). In expansigeny, higher growth of cell walls adjacent to air spaces preferentially enlarges air spaces (purple box in A3 and C).

In plants, air spaces can form by cell separation or cell death. Air space formation by cell death is known as lysogeny [5] and predominantly happens in roots in response to waterlogging, although in some species it also happens in stems and leaves (reviewed in [11,12]). Leaf air spaces largely form via cell separation [13], which can be divided into 2 types—schizogeny, where cells detach from one another [5] (Fig 2A), and expansigeny, where spaces enlarge by selective expansion of cell wall regions adjacent to intercellular spaces [14,15] (Fig 2A). Although both cell death and cell separation are highly regulated to produce reliable air space patterns, air space formation by cell separation is a developmental process that involves sculpting tissue as it grows, whereas air space formation by cell death is a biochemical process that imposes a pattern upon a tissue that has already grown and divided. In this article, I focus on air space formation by cell separation as it is predominant in leaves and the least well understood. For detailed reviews of lysigeny, see [11,16].

Ultimately, air space formation by cell separation is an outcome of differential cell division, expansion, and adhesion. It has been proposed that air space formation is due to localised loss of adhesion at the sites of air space initialisation [17] or to differential growth (combined cell division and expansion) between the epidermis and mesophyll, where greater growth in the epidermis pulls mesophyll cells apart [13]. However, the relative contribution of cell division, expansion, and adhesion has not been experimentally tested and is a key question for future research.

In all species studied, leaf air spaces initially form at multicellular junctions [7,14,17,18] before subsequently expanding. TEM imaging has shown that localised wall breakdown happens at the site of future air space formation in *Phaseolus vulgaris* [17], and modelling of intercellular space formation in xylem fibres suggests that localised loss of adhesion combined with turgor pressure is sufficient to initialise small intercellular air spaces without the need for differential growth between the internal tissues and epidermis [19]. However, it is not clear whether localised changes in cell wall properties are necessary to initialise air spaces or whether stresses due to turgor pressure are highest at multicellular junctions and induce cell separation there despite equal adhesion on all walls. In addition, although this turgor and cell adhesion–driven mechanism can initialise air space formation, it is unable to produce the enlarged air spaces we see in aquatic plants and in the spongy mesophyll of many terrestrial plant leaves. Even if cells continue to expand, once formed, these air spaces would remain a fixed proportion of overall leaf volume as cells remain spheres packed together.

Therefore, to enlarge air spaces, some form of differential growth must be involved, either between tissue layers pulling mesophyll cells apart (schizogeny) or at localised regions of the mesophyll cell wall (expansigeny) (see Fig 2B and 2C). Recent work in *Arabidopsis thaliana* has shown that air spaces in the spongy mesophyll expand by expansigeny [14] and that at early stages of leaf development, growth rates are higher in the epidermis than subepidermis [18] (this differential growth provides the force necessary to pull cells apart in schizogeny). Data from *A*. *thaliana* also suggest that presence of the epidermis is not necessary to form air spaces within the mesophyll, as the *atml1/pdf2* double mutant lacks an epidermis but still forms air spaces between the exposed mesophyll cells [20]. Therefore, it is likely that differential growth both between tissue layers and within mesophyll cells contribute to air space formation in leaves, and work is needed to understand the relative contributions of each by experimentally altering growth differentially between tissue layers or locally within mesophyll cells. Recent advances in microscopy are beginning to enable deeper penetration into tissues, and, now, visualisation of such differential growth patterns in growing leaves is a real experimental possibility. Combining these data with cell-level computational modelling will allow us to understand how such cellular effects control air space formation at the tissue level.

### What molecular mechanisms control and pattern leaf air space formation?

Within a leaf, air spaces are spatially patterned. Aquatic plant leaves often look like waggon wheels in cross section, with enlarged air spaces arranged radially with each separated by a single file of cells (Fig 1A). In terrestrial leaves, air spaces are patterned along the adaxial/abaxial axis, with small spaces between the adaxial palisade mesophyll cells and large spaces between the abaxial spongy mesophyll cells, with particularly large cavities adjacent to stomata.

#### Adaxial and abaxial patterning in air space development

The adaxial/abaxial patterning of mesophyll cell types is controlled by well-known genetic regulators of adaxial/abaxial leaf patterning, including genes from the *HDZIPIII* (adaxial) and *KANADI* (abaxial) families (reviewed in [21]). For example, *A*. *thaliana or Antirrhinum majus* plants lacking adaxial identity form leaves containing only spongy mesophyll cells [22,23]. However, although containing only spongy mesophyll cells, abaxialised leaves contain few, small air spaces [22,23]. This suggests that large air spaces are not simply a product of spongy mesophyll identity. Abaxialised leaves also fail to form a leaf blade and are instead needle shaped, suggesting that expansion of the leaf blade is necessary for air spaces to form in flat leaves and that air spaces may be an emergent property of multicellular leaf growth rather than an intrinsic part of spongy mesophyll cell identity.

These observations suggest that plants localise air space formation to certain regions of the leaf and within certain cell types. However, beyond adaxial and abaxial identity genes, factors that control air space size and arrangement are relatively unknown. This is partly down to difficulties visualising internal tissues and screening for mutants. Indeed, no genes are known to regulate palisade versus spongy mesophyll cell identity and associated air space formation.

Thus far, 2 factors are known to regulate leaf air space patterning: stomatal and chloroplast signalling.

#### Stomatal signalling in air space development

**Fig 3:**
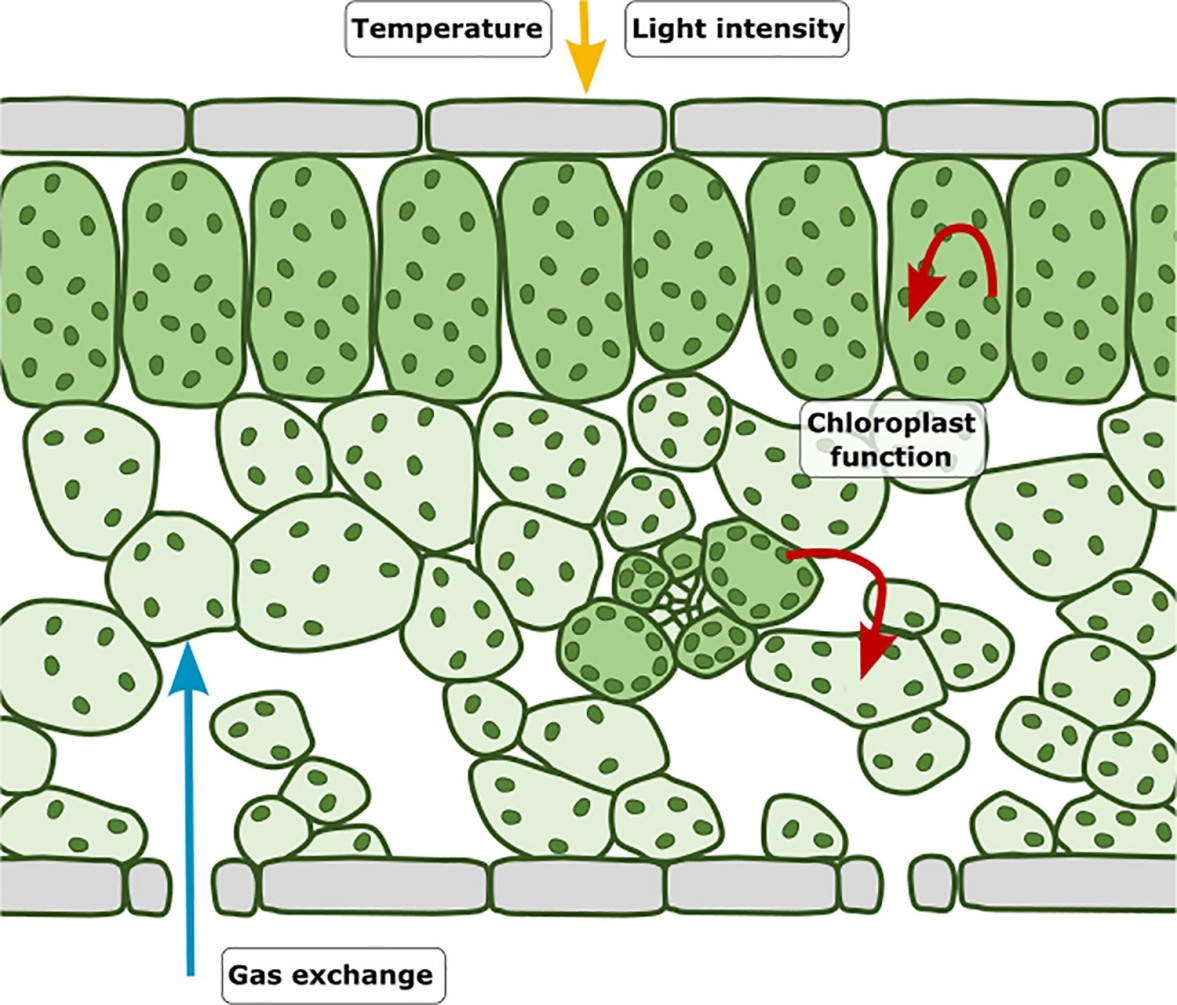
Environmental and physiological signals control air space formation. Gas exchange via stomata promotes air space formation around stomata (blue arrow), chloroplast signalling reduces air space formation throughout the mesophyll and promotes palisade mesophyll identity (red arrows), and light intensity and temperature regulate air space formation, but their effects remain poorly characterised (yellow arrows).

The observation that large air spaces are positioned adjacent to stomata in many species [9,24] (Fig 3) suggests that stomata themselves may regulate the position of large spaces. This has been confirmed by recent work showing that stomatal density and air space volume are positively correlated with both *A*. *thaliana* and wheat [24,25]. Experiments suggest that substomatal air spaces form only adjacent to mature, open stomata [24,25]. For example, in the *A*. *thaliana focl1-1* mutant, stomatal pores are partially occluded by a layer of cuticle, resulting in reduced gas exchange [24]. In these plants, the correlation between stomatal density and air space volume is partially broken, suggesting that the physiological function of stomata may signal to promote mesophyll air space formation. These data do not rule out a molecular signal from mature guard cells to promote air space formation, but they do suggest that air space formation is promoted by the functioning of the open pore itself, likely via gas exchange. The nature of the gaseous signal is still unclear, but the 2 most likely candidates are CO_2_ or H_2_O (water vapour). Future work experimentally altering gas concentrations may be able to identify the gas involved and promises to uncover how the physiological demands of the plant influence development to optimise leaf structure and balance water use efficiency and photosynthesis.

There may also be a role for molecular signals beyond gas exchange, as mutations in the receptor genes *ERECTA* (*ER*) or *TOO MANY MOUTHS* (*TMM*) alter stomatal density and break the observed correlation between stomatal density and air space patterning. This suggests they may provide a molecular link between stomatal and mesophyll development [25,26]. Intriguingly, the mesophyll-expressed STOMAGEN protein is known to move to the epidermis and bind ER and TMM to alter stomatal density [27,28]. This suggests that there may be a feedback loop between the mesophyll and epidermis that fine-tunes stomatal density and air space patterning. However, the interplay between these factors and gas exchange is poorly understood.

#### Chloroplast signalling in air space development

The role of chloroplasts in air space development was demonstrated by analysis of reticulate mutants in *A*. *thaliana*. This class of mutants have pale green leaves with dark green veins. In most reticulate mutants, the pale lamina is caused by a reduction in mesophyll cell density and corresponding increase in air space volume within the leaf (e.g., [29,30]). Most described reticulate mutants are affected in genes that control chloroplast biogenesis or metabolism, resulting in plants with chloroplasts of reduced number and function. These include mutations in the genes *scabra3* [31], *differential development of vascular associated cells 1* (*dov1)* [32], *cab underexpressed1 (cue1)* [33], and *venosa* (*ven*) *3* and *6* [34] (reviewed in [35]). Other reticulate mutations, such as in the *reticulata-related* gene family [29], have no effect on chloroplast development, but the affected genes encode proteins that localise to chloroplasts, further supporting a link between chloroplasts and air space formation.

The observation that leaves with compromised chloroplasts have larger air spaces suggests that chloroplasts signal to mesophyll cells to regulate mesophyll cell proliferation and air space volume. This is supported by data from *Brassica napus* and *A*. *majus* leaves in which chloroplast development is blocked genetically or with spectinomycin (an inhibitor of plastid protein synthesis). Leaves of these plants contain sectors of cells lacking fully developed chloroplasts. In these chloroplast-deficient sectors, palisade mesophyll cells are absent, producing a pale leaf with large air spaces composed largely of spongy mesophyll cells [36,37]. These data suggest a role for chloroplast signalling in both cell identity and air space formation in the mesophyll. However, whether functional chloroplasts signal to enhance mesophyll cell proliferation and reduce air space formation or defective chloroplasts signal to reduce mesophyll cell proliferation and increase air spaces remain unclear [38].

As the above loss of palisade cells is restricted to chloroplast deficient sectors, it is likely that signalling from the chloroplast to regulate palisade identity and air space formation is, at least in part, cell autonomous (Fig 3). However, several of the genes mutated in reticulate mutants are expressed preferentially or exclusively in bundle sheath cells surrounding the vasculature, adding a spatial element to chloroplast regulation of air space patterning [29,39] (reviewed in [35]; Fig 3). This has led to the suggestion that plastids in the bundle sheath may either transmit a molecular signal to regulate mesophyll growth [40,41] or supply necessary metabolites for mesophyll cell growth and division [32]. Any molecular signal is unknown, but the phenylpropanoid-derived secondary metabolite dehydrodiconiferyl alcohol glucoside (DCG) is known to promote cell division and expansion in tobacco [42,43] and is reduced in the reticulate mutant *cue1*, making it a possible candidate [41]. However, its production in and movement from the bundle sheath have not been demonstrated. Other candidates for a possible molecular signal include reactive oxygen species, small interfering RNAs (siRNAs), hormones, other metabolites, or proteins, all of which are known to be mobile and regulate developmental processes. Evidence that the bundle sheath supplies metabolites to the mesophyll is supported by data showing that several reticulate mutants (including *cue1* [41,44] and *ven3* and *6* [34]) are deficient in amino acids and nucleotides, and exogenous application of these metabolites often rescues the phenotype. Further investigation is needed to understand exactly how chloroplasts signal to mesophyll cells to regulate cell identity and growth, but emerging evidence suggests that plastid localised proteins, such as *ENLARGED FIL EXPRESSING DOMAIN 2 (ENF2)* interact with adaxial/abaxial patterning factors to position spongy versus palisade mesophyll cell identity along the adaxial/abaxial axis, providing a tantalising link to well-known regulators of leaf development [45].

#### How does the environment regulate air space formation?

Experimental changes in growth environment suggest that temperature [46], light intensity [25], and shading [47] also regulate air space patterning, but their effects have not been characterised in detail, and the molecular mechanisms by which they act are unknown. It is possible that changes in light and temperature alter stomatal density or chloroplast function, which then alters air space patterning downstream but is also possible that these environmental inputs signal via an independent pathway. Experiments combining environmental perturbations with stomatal and chloroplast mutants can test these hypotheses in the future.

#### How are environmental and molecular signals integrated in air space development?

The data above suggest that the physiological state of the leaf regulates air space formation, via stomata and chloroplast function. However, how these signals are integrated through development to regulate air space formation and patterning is unknown. The literature on chloroplast and stomatal regulation of air space formation are largely separate, so future work analysing higher order mutants with altered chloroplast function and stomatal density is needed to understand how these two regulatory factors interact.

In addition, the question remains of what downstream pathways mediate the effect of stomata and chloroplasts on air space formation. Do they act via the same or different downstream pathways, and what are the molecular factors themselves? How do they regulate expansigeny and schizogeny? Do they regulate mesophyll cell division and expansion, or do they also affect growth in the epidermis to alter differential growth between the epidermis and mesophyll in a more integrated manner? No downstream factors that regulate mesophyll cell division, expansion, and adhesion have been linked to upstream regulators of air space formation, making this a key question for future research. Carefully designed suppressor screens using reticulate mutants as a background may be able to identify downstream genes, and recent advances in single cell sequencing (e.g., [48,49]) open the possibility of directly identifying genes up-regulated in mesophyll cells throughout air space formation. Together, these and other approaches may identify novel regulators of air space formation and start to piece together pathways of regulation.

### What regulates cell expansion, division, and adhesion in air space formation?

**Fig 4:**
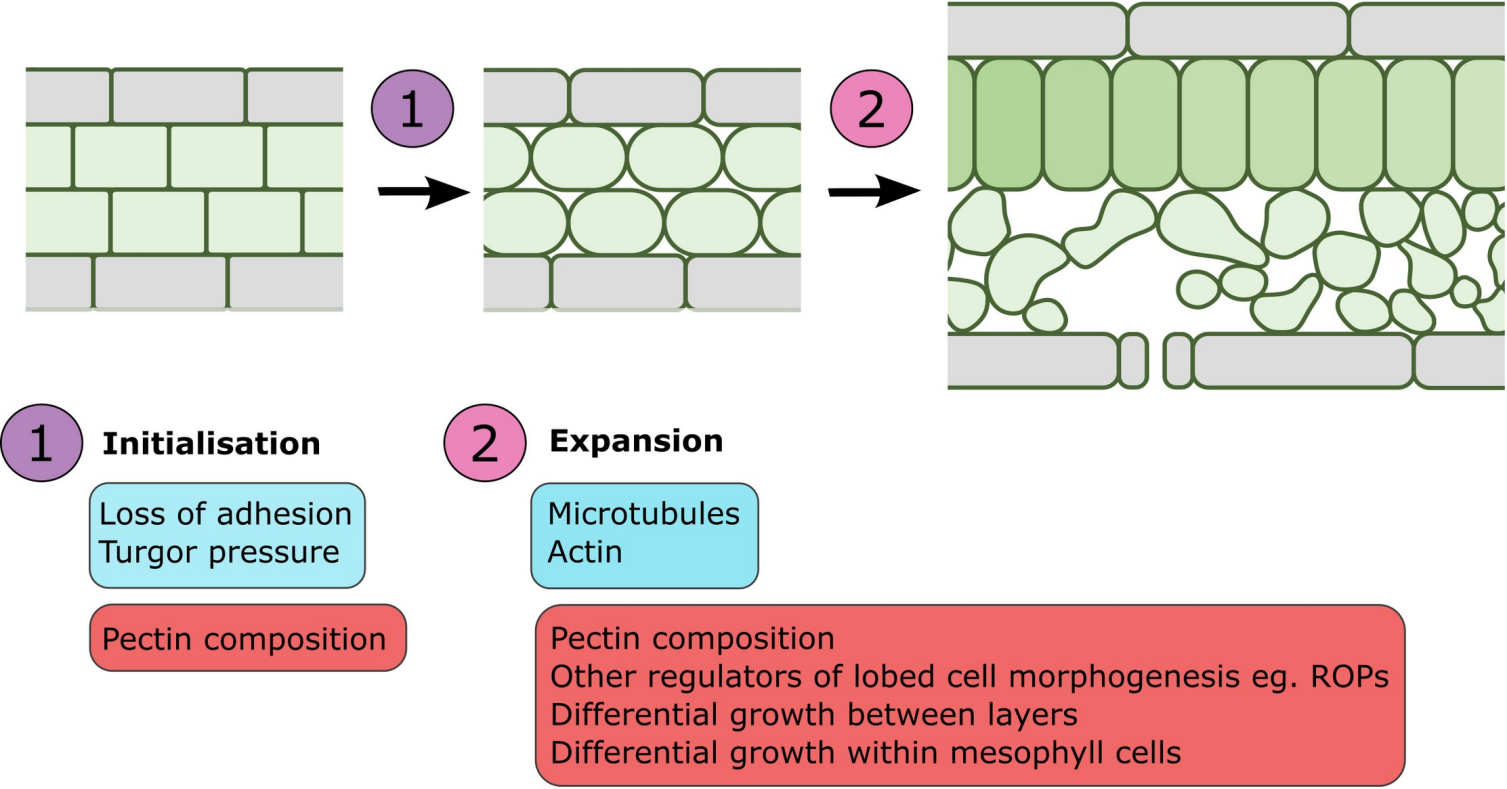
Cellular processes in air space formation. Air space formation can be divided into 2 stages: (1) initialisation; and (2) expansion. In initialisation, a combination of turgor pressure and loss of adhesion causes small air spaces to form at multicellular junctions. In expansion, the actin and microtubule cytoskeletons regulate cellular morphogenesis to promote air space formation. Processes known to control air space formation are highlighted in blue, and processes that have been hypothesised to control air space formation, but remain untested, are highlighted in red.

Downstream of physiological and environmental signals, the molecular mechanisms that regulate cell expansion, division, and adhesion in air space formation, is relatively unknown. Experiments in *A*. *thaliana* have shown that altering cell division in the mesophyll changes air space volume [10], but endogenous regulators of cell division in air space formation are yet to be identified. However, there is emerging evidence that the cytoskeleton may play a role in mesophyll cell morphogenesis and air space expansion, and several genes encoding pectin modifying enzymes have been implicated in cell adhesion, although their role in air space formation is untested (Fig 4). Many genes are also known to regulate lobed cell morphogenesis in the leaf epidermis (reviewed in [50]), but their roles in mesophyll morphogenesis are largely untested, making this a promising avenue for further study.

#### How does the cytoskeleton regulate air space formation?

Work in several species has identified characteristic banding patterns of microtubules during spongy mesophyll cell morphogenesis, where bands of microtubules align opposite each other in adjacent cells [51–54]. This pattern has been proposed to drive air space formation between mesophyll cells via expansigeny by patterning targeted cell wall reinforcement and increasing cell wall growth adjacent to air spaces [51]. This is supported by the observation that leaves treated with the microtubule depolymerising drugs oryzalin and colchicine produce mesophyll cells without lobes and with significantly reduced air spaces that do not expand beyond initial cell separation [51,53–55]. Therefore, microtubules promote air space expansion but not initiation (Fig 4). Because microtubules are key parts of the cell division machinery, it is likely they control both cell division and expansion in air space formation, but the relative importance of each is unclear. It is also an open question how microtubules link to upstream physiological and environmental factors that control air space patterning.

The role of actin in mesophyll cell morphogenesis is less clear. In wheat mesophyll cells, actin filaments form bundles aligned with microtubules [56], and actin depolymerisation by treatment with cytochalasin D prevents microtubule bundle formation and alters mesophyll morphogenesis, suggesting that actin may control mesophyll cell morphogenesis by patterning microtubule bundle formation [57]. However, maize mutants in actin filament organisation contain normal mesophyll cells, despite having epidermal cells with fewer lobes [58]. Therefore, further research is needed to understand the role of actin in air space formation and how it links to physiological and environmental inputs.

#### Do regulators of cell adhesion control air space formation?

In plant cells, intercellular adhesion is controlled by the middle lamella, a pectin rich region between the walls neighbouring cells [59]. Modification of pectin affects its ability to act as glue between cells. Pectin with low levels of methyl esterification promotes crosslinking and adhesion, whereas high levels of methyl esterification reduce adhesion (reviewed in detail in [59]). Localised differences in pectin composition have been described between cell walls in contact with other walls and those abutting an air space in leaves [55,60], but whether these differences cause cell separation or simply reflect different cell wall functions is unknown.

Plants with altered expression of pectin methyl esterase (PME) have altered cell adhesion in leaves [61], and mutants with altered pectin composition show cell adhesion defects in the epidermis [62–64], but any air space phenotypes of these lines have not been characterised, making their role in air space formation unknown. Plants with perturbed cell adhesion often have holes in the epidermis and severe growth defects [65], making the analysis of more subtle phenotypes difficult. The development of conditional lines where cell adhesion is only spatially or temporally compromised will allow a more accurate analysis of the effect of cell adhesion in air space formation.

### How have these mechanisms have been modified to evolve new leaf air space arrangements?

Plants have repeatedly evolved transitions between different air space arrangements—increasing air space size when moving from land to water [4] or reducing the proportion of air spaces when moving to an arid environment [66]. However, the genes that have been modified to mediate these evolutionary transitions are completely unknown.

Mechanisms regulating stomatal density and chloroplast signalling may have been modified through evolution to alter air space patterning, but many aquatic plants do not have stomata on their vegetative leaves despite containing enlarged, highly patterned air spaces [5,6]. This opens the possibility that at least some novel air space arrangements may have evolved by mechanisms that we do not currently understand, perhaps by modifying downstream components that directly regulate cell expansion, division, and adhesion or by evolving entirely new upstream regulators to bypass physiological signalling. The recent development of several aquatic species including *Utricularia gibba* [67–70], *Callitriche pallustris* [71], and duckweed species [72–74] as aquatic plants suitable for experimentation opens the possibility to begin to understand the molecular and developmental basis of how enlarged air spaces evolve. These studies may also allow the identification of factors that regulate air space formation regulators independently of stomata and chloroplast function, which are likely to be less important in aquatic plant leaves.

## Conclusions

The above discussion makes clear that the formation of air spaces in plant leaves is a complex developmental process. Inputs from the environment (light levels, temperature, water, and CO_2_ availability) are sensed at the physiological level within the leaf, and air space formation is altered accordingly to produce a leaf best able to balance photosynthesis and water loss in its local environment. It is unclear how these environmental and physiological signals interact and signal downstream to regulate the cellular processes of cell division, expansion, and adhesion that are necessary to form air spaces, but regulation of the cytoskeleton and composition of the middle lamella are likely to play roles, as is differential growth between tissue layers and within mesophyll cells (Fig 4).

Understanding how these factors are integrated requires work at the juncture of developmental biology, genetics, physiology, and computational modelling. Recent advances in microscopy, including X-ray microcomputed tomography (microCT) and light sheet and 2-photon microscopes, allow penetration into previously inaccessible tissues and are beginning to make imaging the process of air space formation feasible. Improved computing power is also beginning to enable the production of three-dimensional computational models to generate and test hypotheses of how cellular properties control air space formation. Combining these advances with innovative mutant screens and improvements in synthetic biology will allow future work to identify novel regulators of air space formation and test their roles in a targeted manner. This will enable not just the identification of genetic pathways linking environmental inputs to air space formation, but also a mechanistic understanding of how these pathways affect the physical properties of cells and how this, in turn, influences tissue and organ level phenotypes. Understanding the mystery of air space formation promises to not only elucidate a fundamental mechanism of development but may also unlock new ways to alter water use efficiency and photosynthetic efficiency in crops, making this a particularly enticing mystery to solve.

## Abbreviations

*cue1*: *cab underexpressed1*
DCG: dehydrodiconiferyl alcohol glucoside
*dov1*: *differential development of vascular associated cells*
*ENF2*: *ENLARGED FIL EXPRESSING DOMAIN 2*
ER: *ERECTA*
microCT: microcomputed tomography
PME: pectin methyl esterase
siRNA: small interfering RNA
TEM: Transmission Electron Microscopy
*TMM*: *TOO MANY MOUTH*S
*ven*: *venosa*
